# Supplementary material for: Superiority of Mild Interventions against COVID-19 on Public Health and Economic Measures
Source: J Pers Med. 2021 Jul 26;11(8):719. doi: 10.3390/jpm11080719 (PMC8398311; doi:10.3390/jpm11080719)
Supplement: Supplementary file 1 [file jpm-11-00719-s001.zip › jpm-1234338-supplementary.pdf]

Supplementary Materials

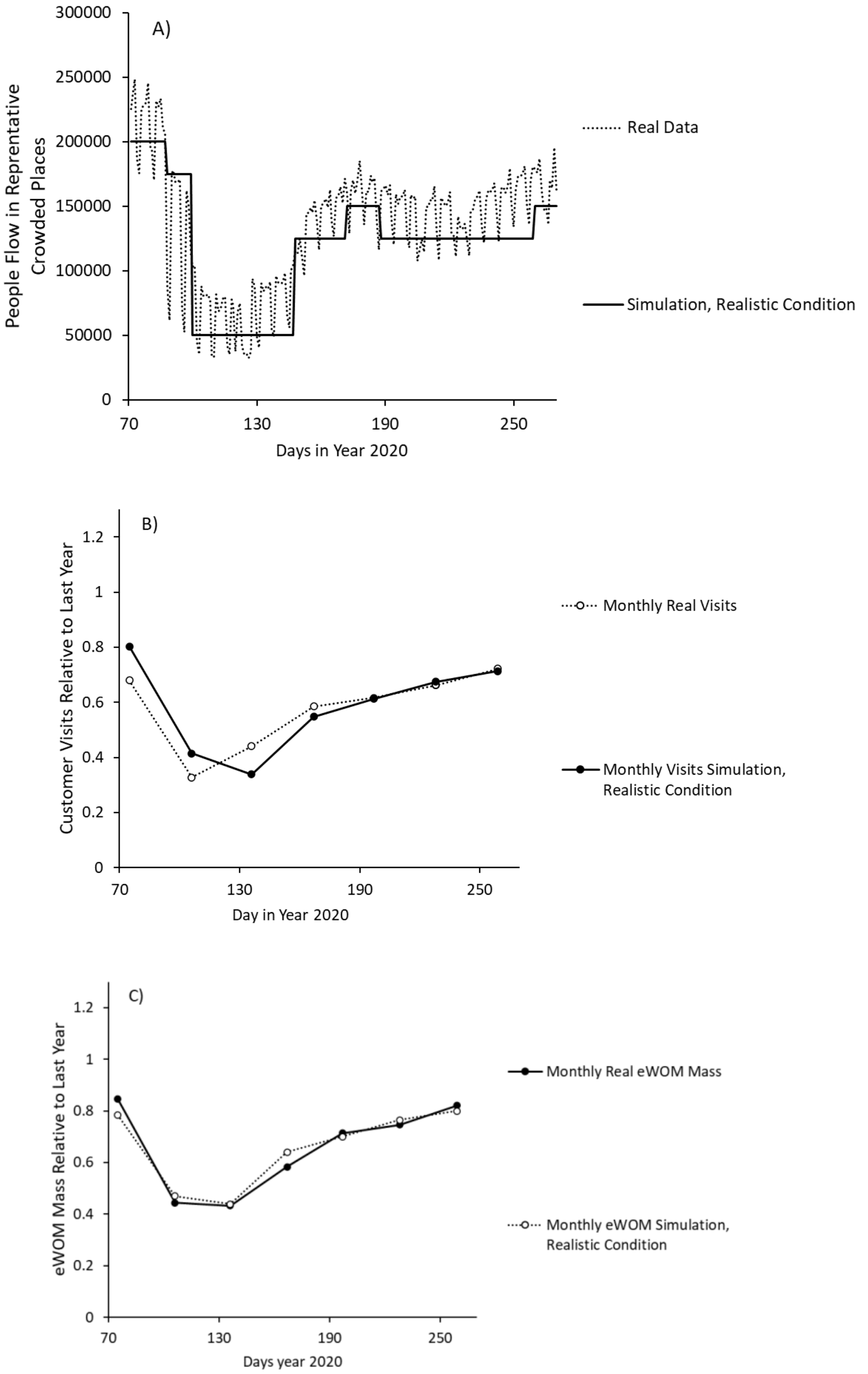

Figure S1. Simulation outcomes for (A) people flow, (B) people dining out, and (C) eWOM mass compared to real metrics.

Table S1. Parametrisation of quantitative model

Parameter	Meaning	Initial value	Equations	Comments
Disease Spreading Part				
Susceptible	Susceptible (not immunised) people	1.40 x 10^7	-Infection	Whole Tokyo metropolitan population
Infected	People newly infected on a given day	149	infection-apparent Infection-inapparent infection	Initial value: calculated from actual confirmed positives after incubation period, apparent ratio, and testing policy
Apparent	People who newly appeared to be symptomatic	60	apparent Infection-not tested-virus testing symptomatic	Initial value: calculated from actual confirmed positives and testing policy
Inapparent	People inapparently infected and acting as virus carriers	664	inapparent infection-inapparent recovery	Initial value: calculated from actual confirmed positives, apparent ratio, and testing policy
Confirmed positives	People tested and confirmed as positive	5	virus testing symptomatic-hospitalisation	Initial value: actual confirmed positives
Not tested positives	People who are symptomatic and treated at home but not virus tested	50	not tested-not tested recovery	Initial value: calculated from actual confirmed positives and testing policy
Susceptible ratio	Effect of probability for a carrier to meet susceptible people	-	Susceptible/1.40 x 10^7	-
Reproduction rate	Standard reproduction rate (2.9 / occasion) calibrated to a unit time (day)	0.207	-	2.9: median of reported R_0 [16]
Temperature effect	Transmission affected by wet balb temperature [18]	1.0	15 May 2020: 1.2<br>15 Jun 2020: 1.6<br>15 Jul 2020: 1.1<br>15 Sep 2020: 1.6	Monthly average temperature was used
Apparent ratio	Apparent ratio	0.375	-	[17]
Incubation time	Incubation time	5	-	[17]
Apparent infection	Daily apparent infections	-	Infected*apparent ratio/incubation time	-
Inapparent infection	Daily inapparent infections	-	Infected*(1-apparent ratio)/incubation time	-
Inapparent virus clearance period	Virus clearance period in inapparent infection	8	-	Tentative turnover 14 days minus infection day and incubation period
Testing policy	Initially, testing frequency was limited	0.5	Raised to 1.0 after 10 May 2020	Half of the fevers are early onset [18]
People Flow and Behavior Part				
Baseline People Flow	Baseline in representative crowded place	250000	-	250,000 per representative traffic node
Daily Maximum People Flow	Maximum people flow in crowded places represented by station	0	Baseline People Flow*(1-0.2 x school closure commuting effect-0.1 x stay-at-home request -0.1 x short-term epidemic consciousness - 0.4 x new normal lifestyle effect	-
Behaviour guidance	Exclusive guidance from Government	0	15 Apr 2020: 1	Recognition at this time point was suggested by LINE survey [31]
Distancing and protective behaviour facto	Reduced risk by individual protective behaviour (wearing masks, distancing)	0.5	-	[15]
Protective behaviour under epidemic condition	Probability for each person to act ideal protective behaviour	0.6	-	Estimated from the finding that about 60% of survey respondents thought more stringent measures were necessary in June 2020 [15]

Protective behaviour under normal condition	Probability for each person to act according to ideal protective behaviours	0.3	-	Half probability of epidemic condition
		Restaurant Industry Part		
Customer Staying home	Potential customer staying home	1.07 x 10^{7}	dining out to home-dining out	Initial value: population of Tokyo consisting of individuals 15 to 74 years of age
Customer Dining Out	Customer dining out	0	dining out-dining out to home	-
Dining Out	Movement to dining out	0	baseline dining out x (1-0.2 x school-closure psychological effect-0.1 x stay-at-home request -0.1 x mid-term epidemic consciousness -0.1 x focused intervention effect -0.3 x long-term epidemic consciousness	-
eWOM Mass	Relative daily eWOM mass	0	(1-0.2 x school-closure psychological effect -0.2 x long-term epidemic consciousness - 0.1 x stay-at-home request -0.1 x focused intervention effect -0.1 x mid-term epidemic consciousness)	-

Table S2. Parametrisation in each intervention scenario

Parameter	Realistic	Second Emergency	Pre-Emptive Shorter Emergency	Exhaustive Emergency
Short-term epidemic consciousness	Initial: 0<br>27 Mar 2020: 1<br>30 May 2020: 0<br>05 Jul 2020: 1<br>15 Sep 2020: 0	Initial: 0<br>27 Mar 2020: 1<br>30 May 2020: 0<br>05 Jul 2020: 1<br>01 Sep 2020: 0	Initial: 0<br>27 Mar 2020: 1<br>30 May 2020: 0<br>28 Jun 2020: 1<br>28 Jul 2020: 0	Initial: 0<br>27 Mar 2020: 1<br>30 May 2020: 0<br>03 Jul 2020: 1<br>01 Sep 2020: 0
Mid-term epidemic consciousness	Initial: 0<br>27 Mar 2020: 1<br>30 May 2020: 0	Initial: 0<br>27 Mar 2020: 1<br>30 May 2020: 0	Initial: 0<br>27 Mar 2020: 1<br>30 May 2020: 0	Initial: 0<br>27 Mar 2020: 1<br>30 May 2020: 0
Long-term epidemic consciousness	Initial: 0<br>27 Mar 2020: 1	Initial: 0<br>27 Mar 2020: 1	Initial: 0<br>27 Mar 2020: 1	Initial: 0<br>27 Mar 2020: 1
School closure, psychological effect	Initial: 1<br>26 May 2020: 0	Initial: 1<br>26 May 2020: 0	Initial: 1<br>26 May 2020: 0	Initial: 1<br>26 May 2020: 0
Stay-at-home request	Initial: 0<br>08 Apr 2020: 1<br>26 May 2020: 0	Initial: 0<br>08 Apr 2020: 1<br>26 May 2020:0<br>19 Jul 2020: 1<br>01 Sep 2020 :0	Initial: 0<br>08 Apr 2020: 1<br>26 May 2020: 0<br>28 Jun 2020: 1<br>28 Jul 2020: 0	Initial: 0<br>29 Mar 2020: 1<br>30 May 2020: 0<br>03 Jun 2020: 1<br>01 Sep 2020: 0
